# A distribution-free convolution model for background correction of oligonucleotide microarray data

**DOI:** 10.1186/1471-2164-10-S1-S19

**Published:** 2009-07-07

**Authors:** Zhongxue Chen, Monnie McGee, Qingzhong Liu, Megan Kong, Youping Deng, Richard H Scheuermann

**Affiliations:** 1 Biostatistics Epidemiology Research Design Core, Center for Clinical and Translational Sciences, The University of Texas Health Science Center at Houston, UT Professional Building, 6410 Fannin Street, Houston, TX 77030, USA; 2 Department of Statistical Science, Southern Methodist University, 3225 Daniel Ave., Dallas, TX 75275, USA; 3 Department of Computer Science, New Mexico Institute of Mining and Technology, Socorro, NM 87801, USA; 4 Department of Pathology, University of Texas, Southwestern Medical Center, 6000 Harry Hines Blvd., Dallas, TX 75390, USA; 5 Specpro, Vicskburg, MS 39180 USA; 6 Department of Biology Science, The University of Southern Mississippi, 118 College Dr., Hattiesburg, MS 39406, USA

## Abstract

**Introduction:**

Affymetrix GeneChip^® ^high-density oligonucleotide arrays are widely used in biological and medical research because of production reproducibility, which facilitates the comparison of results between experiment runs. In order to obtain high-level classification and cluster analysis that can be trusted, it is important to perform various pre-processing steps on the probe-level data to control for variability in sample processing and array hybridization. Many proposed preprocessing methods are parametric, in that they assume that the background noise generated by microarray data is a random sample from a statistical distribution, typically a normal distribution. The quality of the final results depends on the validity of such assumptions.

**Results:**

We propose a Distribution Free Convolution Model (DFCM) to circumvent observed deficiencies in meeting and validating distribution assumptions of parametric methods. Knowledge of array structure and the biological function of the probes indicate that the intensities of mismatched (MM) probes that correspond to the smallest perfect match (PM) intensities can be used to estimate the background noise. Specifically, we obtain the smallest q2 percent of the MM intensities that are associated with the lowest q1 percent PM intensities, and use these intensities to estimate background.

**Conclusion:**

Using the Affymetrix Latin Square spike-in experiments, we show that the background noise generated by microarray experiments typically is not well modeled by a single overall normal distribution. We further show that the signal is not exponentially distributed, as is also commonly assumed. Therefore, DFCM has better sensitivity and specificity, as measured by ROC curves and area under the curve (AUC) than MAS 5.0, RMA, RMA with no background correction (RMA-noBG), GCRMA, PLIER, and dChip (MBEI) for preprocessing of Affymetrix microarray data. These results hold for two spike-in data sets and one real data set that were analyzed. Comparisons with other methods on two spike-in data sets and one real data set show that our nonparametric methods are a superior alternative for background correction of Affymetrix data.

## Introduction

Affymetrix GeneChip^® ^arrays are widely used in biological and medical research to estimate gene expression levels. Each gene is interrogated using 11–20 probe pairs (depending on the platform), each of which consists of a perfect match (PM) and a mismatch (MM) probe. PM probes are sequences of 25 nucleotides that are intended to be a perfect complement to a subsequence of the target transcript of interest (gene). A MM probe is also 25 nucleotides in length, with the same composition as the corresponding PM probe, except that the middle base (13th) is changed to its Watson-Crick complement. The MM probes were originally designed to be different at one base pair so that their intensities could be subtracted from those of the PM as a measure of non-specific hybridization.

In order to estimate gene expression values and perform high-level analyses, such as classification and clustering, probe-level pre-processing of the data is necessary. Typically, there are three steps of preprocessing: background correction, normalization and summarization, although not necessarily in that order. It has been argued that background correction is the most crucial step for probe level processing [1,2]. Thus, it is important to understand the assumptions underlying background correction methods, and test those assumptions, before blindly applying any preprocessing method.

One popular method, Robust Multichip Average (RMA) uses an exponential-normal convolution model for background correction, quantile normalization for the normalization step, and a median polish algorithm to summarize probe level values into a single expression value per gene [3]. Some software packages allow the user to interchange background correction methods with the normalization and summarization methods (*e.g. *Bioconductor [4]).

The exponential-normal convolution model is given by X = S + Y, where X is the observed PM intensity for a probe on the array, S is the true signal, assumed to have an exponential distribution with rate parameter *α*, and Y is normally distributed background noise [3]. The normal noise distribution is truncated at zero so that the model does not return negative intensity values. Background correction involves estimating the parameters *μ *and *σ *of the normal distribution and the rate parameter *α *of the exponential distribution. In practice these parameters cannot be estimated by conventional methods, such as maximum likelihood [1]; therefore, the implementation of RMA background correction in Bioconductor [4] uses an *ad hoc *method. We have previously shown that this method returns poor parameter estimates [5].

**Figure 1:**
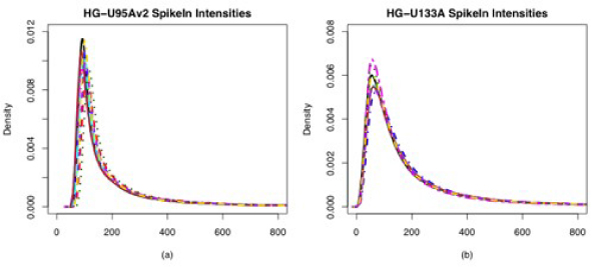
**Smoothed density estimates of raw PM intensities for 10 randomly selected arrays from the Affymetrix Latin Square spike-in experiments**. HG-U95Av2 (a) and HG-U133A (b). Each colored line represents a different experiment. A convolution of a normal distribution and an exponential distribution seem reasonable for these data.

**Figure 2:**
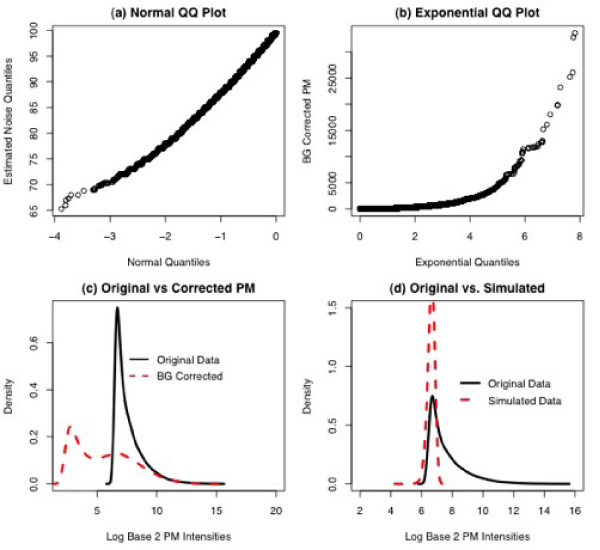
**Quantile-quantile plots and density plots for HGU95 Spike-In data**. (a) Quantile-quantile plot with quantiles of the standard normal distribution on the horizontal axis and quantiles of the noise distribution as estimated by the exponential-normal convolution model. If the normality assumption is correct, the plotted values should lie on a straight line. (b) Quantiles of an exponential distribution versus the background corrected probe-level intensities from the exponential-normal model. Again, any departures from a straight line indicate a lack of fit for the exponential distribution. (c) Density estimates of the log base 2 PM intensities for the original (uncorrected) probe-level intensities (solid line) and the estimated background using the exponential-normal model (dashed line). (d) Density estimates of the log base 2 PM intensities from the original data versus a simulated convolution of a normal distribution and an exponential distribution. The parameters for the normal and exponential distributions were obtained using estimates given by the Bioconductor implementation of RMA.

**Figure 3:**
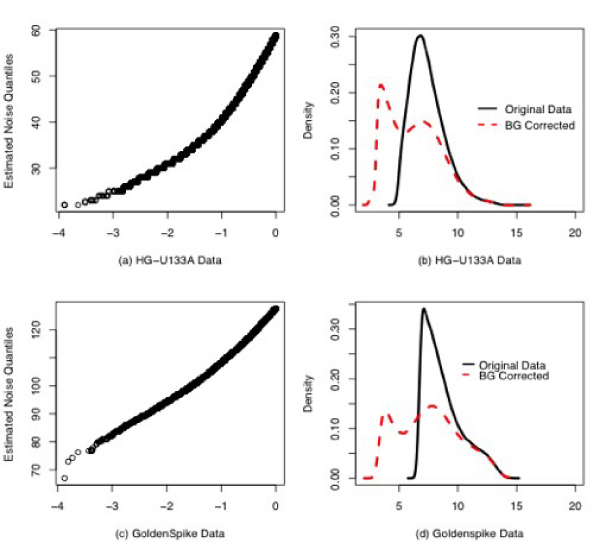
**QQ-plot of estimated background noise (a, c) and density plots (b, d) of original data versus background corrected data for nine arrays from the HG-U133A spike-in data set and the control arrays from the GoldenSpike data set**.

The exponential-normal convolution model is built on the reasonable assumption that fluorescence intensities from a microarray experiment are composed of both signal and noise, and that the noise is ubiquitous throughout the signal distribution. A convolution model of a signal distribution and a noise distribution is a natural choice in such a situation. The choice of a normal distribution for the background noise and an exponential distribution for the signal was likely made for two reasons. First, density estimates of raw PM intensities from the Affymetrix Latin Square spike-in data sets show a right-skewed curve with what looks like a long exponential tail (see Figure 1). Second, the normal and exponential distributions are easy to manipulate mathematically in order to obtain a closed form for the expectation of the signal given the observed values, which is necessary for parameter estimation. However, Figures 2 and 3 in the next section show that the convolution of a normal and an exponential distribution is not generally a good fit for microarray data. These observations, plus the difficulty of checking assumptions and estimating parameters, motivate a nonparametric background correction method.

The objective of this paper is to introduce a new background correction method, called Distribution Free Convolution Model (DFCM). The model has the same form as the exponential-normal convolution model (X = S + Y), except that no distributional assumptions are made on the signal (S) of the noise (Y). The mean and variance for the noise distribution are estimated using MM probe intensities in a novel way that is more consistent with their biological and structural characteristics. The signal is given by the PM intensities once the estimated background has been subtracted (as explained in Methods); therefore, there is no need to estimate parameters for the signal. We compare DFCM against RMA, RMA with no background correction (RMA – noBG) [6], GCRMA [7], MAS 5.0 [8], dChip [9,10], and PLIER [11]. In general, DFCM outperforms these other methods for two different spike-in data sets and a real data set involving the role of CD40 in regulatory networks in human B cells [12].

## Methods

### Estimating the distribution-free convolution model

Let X = S + Y, where X = observed PM intensities, S = true intensity signal, and Y = background noise. The DFCM algorithm for background correction proceeds as follows:

1. Obtain the lowest q1 percent PM intensities. q1 is typically a small number (less than 30%). Information on the estimation of q1 is given in the next section.

2. Obtain lowest q2 percent (typically 90% or 95%) of MM intensities associated with the PMs obtained in step 1. These MM intensities are a measure of background noise, and will be termed "noise" in the sequel.

3. Use a nonparametric density estimate of the lowest q2 percent of the MM intensities to find the mode of the noise distribution. By default, the DFCM algorithm uses an Epanechnikov kernel estimate. Consider this mode as an estimate of the mean of the noise distribution. We denote this estimate as .

4. Estimate the standard deviation of the background noise by calculating the sample standard deviation of the noise for values that are smaller than . Then  is the sample standard deviation multiplied by . The square root of 2 enters into the estimation of sigma due to the use of only one side of the noise distribution (those values less than the mean) to estimate the standard deviation.

5. Obtain background-corrected PM intensity values for the *k*^*th *^probe (k = 1,..., K) in the *i*^*th *^probe set (gene), , using the following formula, where *min *denotes the minimum intensity (PM or MM). Here, *x*_*ki *_denotes non-corrected PM intensity values. Let



Therefore, for large enough values of *x*_*ki*_, we correct for background by subtracting the estimated mean of the background noise. For small values of *x*_*ki*_, the background corrected PM intensities are determined by a linear interpolation, where the slope is a function of the background mean and standard deviation. The two equations will give the same result if .

After background correction, any method of normalization or summarization can be used. For the purposes of this paper, quantile normalization and median polish summarization are used for DFCM.

### Choosing q1 and q2

Background noise is estimated using a select set of MM probe signals that are not likely to include effects of non-specific hybridization to the PM target transcript or cross-hybridization to a related target transcript. In choosing *q*_1 _and *q*_2_, we are attempting to choose PM values that are small enough so that non-specific MM hybridization to a PM target is not a problem, and then choosing a subset of MM values that are unlikely to be affected by cross-hybridization.

The parameter *q*_1_can be thought of as a measure of the percentage of PM probes that recognize genes that are not expressed in the data set, based on the assumption that in any given biological sample there will be a subset of genes that are not expressed at a detectable level. Any signal detected for MM probes for these genes cannot be due to non-specific hybridization because the gene is not expressed, based on the low PM values.

To choose the parameter *q*_1_, we developed an algorithm which calculates *q*_1 _such that the proportion of MM intensities greater than the PM intensities for the smallest *q*_1_% of the data is approximately 50%. We believe that one of the reasons that MM intensities are sometimes greater than their corresponding PMs is non-specific hybridization. Therefore, in estimating *q*_1_, we obtain a measure the percentage of non-specific hybridization in the data set.

### Data sets used for comparison

The two Affymetrix Latin-Square spike-in data sets (HG-U113A and HG-U95Av2) each contain several spiked-in transcripts in known locations on a set of chips. These data sets, and a detailed description of the Latin Square design are available at . Affymetrix has reported that certain probe pairs for transcripts 407_at and 36889_at had been found to perform poorly in the HG-U95Av2 spike-in data. In addition, other researchers have found that the number of spike-in probe sets should be 16 instead of 14. Two articles [6,13] report that probe set 546_at should be considered with the same concentration as 36202_at since both of them were designed against the target Unigene ID Hs. 75209. Further, probe set 33818_at should be included as a spiked transcript in the 12th column of the Latin square design. Our definition of spike-ins for the HG-U95Av2 data includes all four of the above mentioned probes, resulting in a total of 18 spiked-in transcripts.

The HG-U133A experiment differs from the HG-U95Av2 experiment in several important ways. First, the HG-U133A experiment consists of 42 specific transcripts that are spiked in at 14 concentrations ranging from 0 pM to 512 pM, again arranged in a Latin Square design. Therefore, there is a finer gradation of concentrations used than in the HG-U95Av2 experiment. Also, there are three transcripts spiked-in at each concentration and three replicate arrays for each experiment, thus a total of 42 arrays. For convenience, we will call the triples of probe sets that recognize transcripts spiked-in at the same concentration "groups".

Recently, the HG-U133A data has also been examined for the presence of additional spike-ins [14]. Twenty-two additional spiked-in transcripts were found. Most of the "new" spike-ins are variants of the original spike-in probe sets, or share a large percentage of probe sequences in common with original spike-ins. For example, the probe sets initially described as recognizing bacterial controls (*e.g*. AFFX-LysX-3_at, AFFX-DapX-3_at, AFFX-PheX-3_at) are targeted at the 3' end of the gene (hence the notation "-3" in the name of the probe set). It makes sense that the probe sets recognizing the 5' and middle sections of the same genes would behave as spike-ins, since the target RNA mixture for hybridization is likely to be made up sequences covering the 5' end and middle regions of the genes. Indeed, the use of 22 additional spike-ins in Receiver Operating Characteristic (ROC) curve plots and Area Under the Curve (AUC) calculations improved the sensitivity and specificity of RMA, RMA with no background correction (RMA-noBG), MAS 5.0, PLIER, and dChip. GCRMA performed slightly worse with the use of all 64 spike-ins. For the ROC curves and AUC calculations that follow, we use a total of 64 spike-ins for the HG-U133A data (42 original spike-ins plus 22 "new" spike-ins)..

We use a third spike-in experiment to examine the distributional assumptions of the exponential-normal convolution model [2]. This series of spike-in experiments was run on the DrosGenome1 chip, and has been named the GoldenSpike experiment. In addition to targeting a different organism than the Affymetrix spike-in data, the GoldenSpike experiment contains 1331 spiked-in transcripts whose levels are varied and 2,551 RNA species whose levels are held constant between the control and test array sets. The large number of spiked-in transcripts allows for more accurate estimates of the false positive and false negative rates and provides an RNA mix that more closely resembles total cellular RNA. Furthermore, no transcript targets were included for approximately two-thirds of the probe sets, allowing for an accurate definition of background data. In contrast, Affymetrix uses an uncharacterized RNA background for their spike-in data sets. Lastly, the fold differences between the test and control array sets for some of the spike-in transcripts are very low (1.2 fold), which allows an estimate of the reliability and sensitivity of detection of small fold differences.

While this data set was used to examine the distributional assumptions of the exponential-normal convolution model, these data were not used for evaluation of the relative performance of DFCM versus other algorithms due to controversy surrounding the use of the GoldenSpike dataset for method comparison. It has been observed that the GoldenSpike experiment uses technical replicates of a single experiment, rather than biological replicates. Thus, random variability in the experiment is confounded with real signal [15]. Others have found that features spiked-in at a 1:1 ratio tend to have different behavior for the control and spike-in experiments [16]. For these reasons, we restricted our comparisons of ROC curves and AUC calculations to the two Affymetrix Latin Square data sets.

### Examining distributional assumptions

In order to test the validity of the noise and signal distributional assumptions, we compared background noise distribution estimated by the exponential-normal convolution model with the standard normal distributions in both quantile-quantile (QQ) plots and density plots using the Affymetrix Latin Square spike-in data sets. All calculations were done using the Bioconductor suite in the R software package for statistical analysis [4].

Quantile-quantile (QQ) plots are designed to compare the distributions of two data sets usually a "gold standard" and a test data set. Sometimes, the gold standard consists of simulated values from a distribution of interest (*e.g. *the normal distribution), and sometimes it is simply data observed from another experiment. If the gold standard is simulated from a known distribution, the purpose of the plot is to see if the observed data have that particular distribution. The sorted values for one data set (quantiles) are plotted on the horizontal axis, and the sorted values of the other data set on the vertical axis. If the plot results in a straight line, then this is evidence that the two data sets have the same distribution.

We also examined the assumption of a normal background distribution using three normality tests: Shapiro-Wilk, Anderson-Darling, and Kolmogorov-Smirnov [17,18], as implemented by the R software package [19]. For each of the spike-in data sets, the background noise was estimated using the Bioconductor implementation of RMA background correction [4]. Once the noise vector was estimated, a random sample of length 100 was taken and the tests were applied to this vector. This was done because normality tests can be extremely sensitive to sample size, often rejecting the null hypothesis of normality just because the sample size is extremely large. A sample size of 100 is large enough to have reasonable power against some alternatives, but not so large that the tests would reject in error [20]. The samples were submitted to each of the three tests 1000 times, and the p-values for each iteration recorded. The results are given in Table 1.

**Table 1:** Results of the tests of normality of the background noise as estimated by the exponential-normal convolution model.

Data Set	Test	Rejection Rate	Min P-valuea	Med P-value	Max P-value
HG-U95Av2	AD	962	0	0.0008	0.4738
	KS	796	0	0.0082	0.8261
	SW	999	0	0.0036	0.1186
HG-U133A	AD	850	0	0.0064	0.5915
	KS	594	0	0.0307	0.7700
	SW	962	0	0.0031	0.3010
GoldenSpike	AD	885	0	0.0035	0.3559
	KS	639	0	0.0259	0.7490
	SW	987	0	0.0016	0.1502

The rejection rates (number of p-values less than 0.05) are much higher than expected, indicating that the background noise is not likely to be normally distributed.

a P-values are not identically 0, but are 0 to at least five decimal places.

**AD **= Anderson-Darling test, **KS **= Kolmorgorov-Smirnov test, **SW **= Shapiro-Wilk test.

### ROC curves and Area Under the Curve (AUC)

In order to compare the performance of DFCM versus currently available methods, we examined ROC curves and AUC for the two Latin Square spike-in data sets mentioned previously. We tested the performance of DFCM against RMA, RMA-noBG, GCRMA, MAS, dChip, and PLIER. All data files were preprocessed together for each method. For the Affymetrix data sets, we compared pairs of experiments that were separated by the same number of permutations of the Latin Square (where d = number of permutations), and obtained average true and false positive rates for each preprocessing method for each value of d, d = 1,..., 7. In these Latin Square designs, d can be thought of as the log2 fold difference in spike-in transcript levels for a majority of the transcripts. For example, for the HG-U133A data set, experiments 1 and 2, 2 and 3, 3 and 4, *etc. *are separated by one shift in the Latin Square design; therefore, d = 1 for these pairs. For twelve groups of spiked-in transcripts (there are three spike-in transcripts per concentration group in the HG-U133A experiment) in each of these fourteen pairs of experiments, there is a 2-fold difference in concentration. Similarly, experiments 3 and 5, 4 and 6, and 5 and 7 are separated by two permutations in the Latin Square design; therefore, d = 2. Eleven spike-in groups have fold changes of 2 on the log base 2 scale between pairs of experiments.

We compared experiments with d = 1 through d = 7, since d = 8 is equivalent to d = 6, d = 9 equivalent to d = 5, and so on. AUC calculations were done for a cutoff of 100 false positives for the HG-U95Av2 experiment, and 200 false positives for the HG-U133A experiment. These cutoff points correspond to a false positive rate of approximately 0.8% for both experiments.

Again, the GoldenSpike data was not used for methods comparison due to serious design flaws, described fully in [15,16].

## Results

### Testing distributional assumptions for the convolution model

In order to test the validity of the noise and signal distributional assumptions, we compared background noise distribution estimated by the exponential-normal convolution model with the standard normal distribution in both quantile-quantile (QQ) plots and density plots using the Affymetrix spike-in data sets. All calculations were done using the Bioconductor suite in the R software package for statistical analysis [4]. R code is provided in Additional file 1.

Figure 2a shows a QQ plot of the estimated background noise for four randomly selected experiments (and their replicates, for a total of 12 arrays) from the HG-U95A spike-in data. The plot is given on the original scale, since the assumption of normal background noise is applied to the probe-level intensities on the original scale. The background was estimated using the RMA background correction method as coded in the affy package of Bioconductor [4]. According to the assumptions of the exponential-normal convolution model, the background noise should have a truncated normal distribution. Therefore, a plot of the background noise estimated using the convolution model versus values simulated from a truncated normal distribution should produce a straight line. In Figure 2a, there are several values deviating from a straight line in the lower left corner of the graph, and the line is bent slightly. However, both of these departures are small. For this data set, assumption of normality for the background noise seems to be reasonable.

Figure 2b is a QQ plot of the background corrected PM intensities (on the original scale) versus quantiles from an exponential distribution for the same data set. The rate parameter used for the exponential distribution is equal to the estimated rate parameter of the signal given by the affy package. The QQ plot for the background corrected (signal) intensities does not show a straight line; in fact, it shows that the distribution of the signal is much heavier tailed than one would expect if the data were exponentially distributed. This suggests that either the exponential model is not a good one for the signal from the PM intensities, or the background correction algorithm is flawed. Indeed, given the heterogeneity of the variances for the intensity level of each gene, we would not expect a clean fit to any distribution, which further bolsters our argument for the application of a non-parametric background correction method.

Figure 2c shows density estimates of the observed log base 2 PM intensities (solid line) and the same intensities after background correction with the exponential-normal convolution model (dashed line). The background corrected intensities should exhibit an exponential distribution. However, the signal from these data has two modes, suggesting that the estimated signal is composed of a mixture of two or more distributions rather than a single exponential distribution, at least for this data set. This density estimate suggests that there are two groups of genes in this data set – genes that are expressed at low levels, and fewer genes expressed at higher levels.

Figure 2d shows the same density estimate of the original PM intensities that was seen in plot 4c, but now this density is plotted against a density consisting of a simulated convolution of a truncated normal and an exponential, using parameters estimated by the background correction algorithm given in Bioconductor. The parameters for the normal and exponential distributions were obtained using estimates given by the Bioconductor implementation of RMA. The estimation procedure for the convolution model produces a decent estimate of the mean, but is not accurate for the rate parameter.

The results shown for the HG-U95Av2 spike-in data apply to the HG-U133A spike-in data, with one notable exception. Figure 3a shows the QQ plot for 3 randomly selected experiments (and their replicates, for a total of nine arrays) from the HG-U133A experiment. Clearly, the background as estimated by the exponential-normal model does not have a normal distribution, since the QQ plot does not display a straight line. In addition, once the data are background corrected, the resulting distribution is not exponential (Figure 3b). Figure 3c shows a QQ plot of estimated background noise data from the GoldenSpike experiment [2]. This plot seems to support a normally distributed background, but not an exponentially distributed signal (Figure 3d).

Table 1 gives the results of the three tests of normality for the estimated background noise for all three data sets. Tests were done using probe-level data on the original scale. We calculated the number of p-values that were less than 0.05, in order to ascertain how often each test rejected. If the null hypothesis were true, we would expect rejections approximately 5% of the time. For all of the results, the rejection rates are much higher, indicating that the data are not at all normally distributed. We also give the minimum, median, and maximum of the 1000 p-values calculated for each test. The minimum p-values are all 0 to at least five decimal places. Median p-values are typically less than 0.001, again indicating that the tests reject often. The fact that the low power KS test at n = 100 yields such a preponderance of small p-values is convincing evidence against normality.

### Downstream performance of DFCM

The quantile-quantile plots provide evidence that the exponential-normal convolution model does not fit the data. These observations lead us to develop the DFCM as a means of background correction that does not rely on specific distributional assumptions. The estimation of noise and signal using DFCM is described in the Methods section. In order to determine if DFCM leads to an improvement of background correction and signal estimation, we applied DFCM and the other methods to each of the Affymetrix Latin Square data sets, and evaluated their performance characteristics using ROC/AUC analysis. The GoldenSpike data set was not used for this comparison because of the controversy around this data set [15,16]. Once the background is corrected using DFCM, the data are normalized using quantile normalization and summarized with median polish.

In order to compare the performance of DFCM with other commonly used methods, we examined ROC curves and AUC analyses for the two Latin Square spike-in data sets mentioned previously. We tested the performance of DFCM against RMA, RMA-noBG, GCRMA, MAS, dChip, and PLIER. All data files were preprocessed together for each method using a PowerMac G5 running R Cocoa GUI with R version 2.8.1 [21]. For the Affymetrix data sets, we compared pairs of experiments that were separated by the same number of permutations of the Latin Square (where d = number of permutations), and obtained average true and false positive rates for each preprocessing method for each value of d, d = 1,..., 7. A more detailed description of the parameter d is given in the methods section.

**Figure 4:**
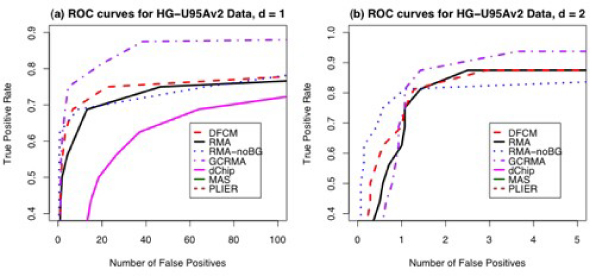
**ROC Curves for HG-U95Av2 Latin Square Spike-In Experiment for d = 1 (a) and d = 2 (b)**. For other values of d, the differences among methods are very small. Curves for MAS 5.0 and PLIER (and dChip for plot b) do not appear on the graphs because their false positive and true positive rates are too small for the scale given. The scale goes from 0.4 to 0.9 in order to magnify differences among the methods.

Figure 4 shows the ROC curves generated from results of analysis to identify differentially expressed genes using various methods on the HG-U95Av2 spike-in data. For Figure 4a, d = 1, and in Figure 4b, d = 2. In this case, q1 = 30% and q2 = 90% for DFCM. For these spike-in data sets, true positive and false positive results can be determined based on the nature of the Latin square design. DFCM and GCRMA both perform well for this data set based on AUC analyses.

**Figure 5:**
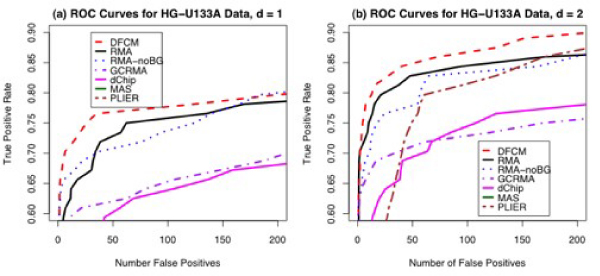
**ROC Curves for HG-U133A Latin Square Spike-In Experiment for d = 1 (a) and d = 2 (b)**. For other values of d, the differences among methods are very small. The lines for MAS 5.0 and PLIER (for plot a) and MAS 5 (for plot b) do not appear on the graphs because their false positive and true positive rates are too small for the scale given. The scale goes from 0.6 to 0.9 in order to magnify differences among the methods.

Figure 5 shows the ROC curves generated from the HGU133 data for d = 1 (a) and d = 2 (b). For these data, DFCM outperforms all versions of RMA. Recall that the normal distribution is not a good fit to the background noise as estimated by RMA (Figure 3a). In this case, a nonparametric approach works better because there is no distributional assumption on the background. Other contributing factors could be larger number of the spike-in transcripts (64 for the HGU133 data versus 16 for the HGU95 data), and the different chip platform. Since the exponential-normal convolution model was developed before the HGU133 spike-in data was available, it may be the case that the model was optimized to perform well on the HGU95 spike-in data.

Table 2 reports the average AUC values for these methods based on two spike-in data sets and different values of d. The results show that DFCM performs best for most of the time. For example, except for d = 2, DFCM outperforms all other methods on the HGU95 spike-in data. For HGU133 spike-in data, DFCM has the largest AUC values for all situations except d = 4.

**Table 2:** Average AUC for RMA, RMA-noBG, MAS 5.0, dChip, and PLIER for detection of spiked-in genes in the Affymetrix Latin Square spikein experiments, according to the value of d, which is related to the log base 2 fold change between experiments.

d	DFCM	RMA	RMA-noBG	MAS 5	dChip	PLIER
Average AUC for the HG-U95Av2 Latin Square Spike-In Data Set						

1	**0.732**	0.715	0.721	0.063	0.572	0.062
2	0.871	0.869	**0.918**	0.167	0.803	0.316
3	**0.936**	0.935	0.935	0.484	0.886	0.629
4	**0.997**	0.994	0.986	0.798	0.948	0.769
5	**1.000**	0.999	0.999	0.916	0.980	0.853
6	**1.000**	**1.000**	**1.000**	0.967	0.987	0.876
7	**1.000**	**1.000**	**1.000**	0.981	0.999	0.876
Average AUC for the HG-U133A Latin Square Spike-In Data Set						
1	**0.768**	0.738	0.734	0.060	0.600	0.365
2	**0.858**	0.831	0.812	0.307	0.709	0.752
3	**0.935**	0.904	0.908	0.561	0.811	0.883
4	0.934	**0.964**	**0.964**	0.837	0.913	0.951
5	**0.983**	0.990	0.983	0.939	0.971	0.985
6	**0.999**	0.998	0.996	0.968	0.989	0.994
7	**0.999**	**0.999**	**0.999**	0.978	0.972	0.996

Larger values of d correspond to larger fold changes among spiked-in transcripts between pairs of experiments. Eighteen spike-in probes sets were used for the HG-U95Av2 experiment, and 64 spiked-in probe sets were used for the HG-U133A to calculate true and false positives, as discussed in Methods. To calculate the AUCs, the number of false positives was set to 100 for the HG-U95Av2 experiment, and 200 for the HG-U133a experiment. Numbers in bold indicate the best value for each row.

### Clustering and classification comparisons using real data

It has been argued that comparisons based on spike-in data do not necessarily translate to data derived from real biological specimens [22]. Therefore, we applied Gene Ontology to validate our result based on the premise that any improvement during the microarray data analysis process should result in tighter clustering of functionally related genes [23]. For example, in a gene list of size g, suppose that f number of genes are annotated with a given GO term. Suppose further that, after clustering the gene list using an accepted clustering method, n numbers of genes annotated with the given GO term are co-clustered together in a cluster with c number of genes. The probability of this specific GO term co-clustering can be calculated based on a hypergeometric distribution [24], and has been implemented in the CLASSIFI website . Smaller probabilities indicate that the clustering is less likely to be due to chance. Therefore, we would expect that the preprocessing method producing the smallest GO term co-clustering P-values would be the method that most effectively reduces noise in the data.

Table 3 shows the number of GO terms with p-values less than 10-10 for each of the combinations of background correction and normalization algorithms tested. The data used were selected from the GSE2350 series [12], downloaded from the NCBI GEO database . In the comparison, the first three samples from both "control" (GSM44051, GSM44052 and GSM44053) and "CD40L treatment" (GSM44057, GSM44058 and GSM44059) groups are used. DFCM outperforms the others when paired with scale normalization, and performs comparably to the others when paired with loess normalization. The zonal background adjustment as given in MAS 5.0 has the overall worst performance. We can also make a case that quantile normalization gives the worst results of the normalization methods presented here.

**Table 3:** Number of GO terms with p-values less than 10^-10 ^for four pre-processing algorithms, according to CLASSIFI on the GSE2350 data. Larger numbers indicate better performance.

Normalization	Background Correction Methods			
	
	DFCM	RMA	None	MAS 5
Loess	86	87	88	57
Quantile	48	47	50	60
Scale	83	80	76	24

To examine the effect of normalization on the results, quantile normalization, scale normalization (as defined for the MAS 5.0 algorithm) or loess was used in combination with each of the background methods discussed in this paper. All methods (except for MAS 5.0) used median polish summarization. Differentially expressed genes were selected using two-sample t-tests. The methods GCRMA, dChip and PLIER could not be used because their background correction, normalization, and summarization algorithms cannot be separated easily.

## Discussion

The RMA convolution model for background correction of microarray data from Affymetrix platforms is very popular. This model assumes that the observed value of fluorescence intensities is composed of an exponentially distributed signal with underlying normally distributed noise. This idea of a combination of signal and noise is quite reasonable, but the analysis presented here indicates that the distributional assumptions are not always correct. In order to examine the assumption of normally distributed background noise, we performed background correction using the convolution model and plotted the estimated background intensities versus a normal distribution using a quantile-quantile plot for three spike-in data sets. The plots indicate that the normality assumption may not hold for all of the spike-in data sets examined. To confirm this, we examined the data with three well-known goodness-of-fit tests. The KS test, in particular, is known to have extremely low power [20]. The fact that the test rejects so often is quite strong evidence against normality.

We also examined the background corrected intensities, which are purported to represent the true signal, against the exponential distribution. QQ plots and goodness of fit tests show that the background corrected signal is clearly not exponentially distributed for any of the data studied here. These observations lend credibility to the notion that preprocessing approaches should not rely heavily on distributional assumptions.

There is some evidence that the gene distributions within groups are normally distributed after preprocessing with MBEI and MAS 5.0 [25]; and these distributions are indeed relevant for the purposes of testing the differential expression of genes with parametric methods such as the t-test. However, we are concerned in this paper with the distribution of the background noise, and not with the distribution of individual probes. In our framework, the background noise results from a combination of autoflourescence (a constant) and non-specific hybridization. As non-specific hybridization is not likely to be gene (and thus probe) specific, it is reasonable to model it with a global distribution [26].

Recently, it has been argued that the assumption that intensity values from a microarray study are random samples from any statistical distribution is seriously flawed [27]. The notion of a random sample implies independence of the intensity values, or at least that the dependence structure is sufficiently weak so that the random sample assumption is plausible. However, the dependence structure among genes, and the probe sets that interrogate them is quite complicated and, in some cases, strong. In this light, a nonparametric approach to background correction is a good alternative. DFCM does not make any assumptions on the dependency structure of the PM or MM intensities. This is manifested in the fact that linear interpolation is used to correct for background with small intensities. With larger intensities, the estimated background mean is simply subtracted because the impact of background noise is minor for the larger intensities.

DFCM uses q2th percentile of the MM signal corresponding to the smallest q1 percentage of PM intensities to estimate background noise. The original intent of the MM probes was to provide a measure of non-specific hybridization that could be subtracted from the PM intensities, leaving the true signal. MAS 5.0 was developed under this assumption. It should be noted that the use of PM values alone could be justified by the noisiness and lack of validity of the MM measurement [3]. For example, approximately one-third of the MM intensities are greater than their corresponding PM intensities, and this tends to be constant across all Affymetrix platforms [3]. Furthermore, the MM measurements tend to be highly correlated with the corresponding PM measurements, indicating that the MM probes are either cross hybridizing to the incorrect gene or non-specifically hybridizing to the correct gene. Therefore MM probes are imperfect predictors of non-specific binding [28]. Thus, PM signal correction through MM subtraction has been largely rejected in the field.

There is a biochemically defensible rationale for estimation of background noise using DFCM. By selecting the lowest q1 percent of PM, we ensure that non-specific hybridization will not be an issue. However, there could still be some cross-hybridization, which is eliminated by taking only the smallest q2 percentage of MM. Having said this, one could simply use the lowest q1 percent of the PM; however, selecting the value for q1 could be difficult and somewhat arbitrary, and if a relatively high value for q1 is chosen might include some real signal for some of the PMs. The corresponding MMs in this case should be less and should be closer to background since in theory they should not be hybridizing to the real target.

The algorithm for choosing the value of q1 is very stable (see Methods), almost always choosing the same value of q1 for a given platform. For example, two experiments completed on the HGU95 platform will have very similar values of q1 (approximately 0.25). In other words, the values of q1 are more platform dependent than they are experiment-dependent. This fact supports the notion that different normalization procedures are required for different platforms [22].

**Figure 6:**
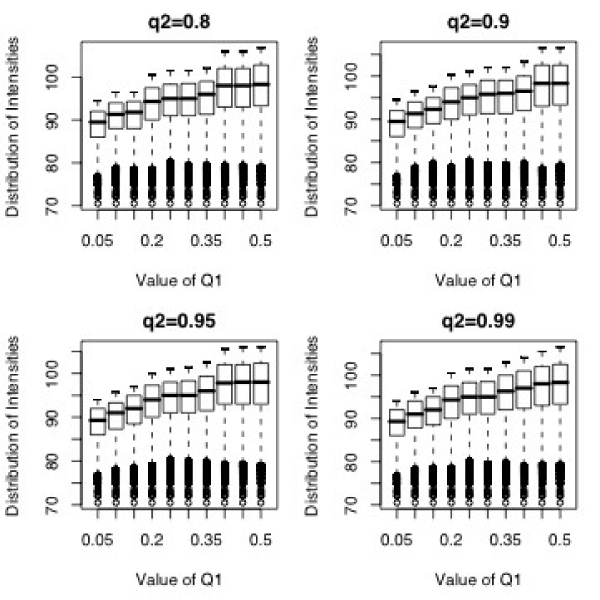
**Boxplots for MM intensities for q1 ranging from 0.05 to 0.5, and q2 = 0.80 (left top), 0.90 (right top), 0.95 (left bottom), and 0.99 (right bottom)**. Our algorithm determined 0.25 to be the optimal value for q1. The value of q2 does not appreciably change the distribution of the MM intensities.

One way to think of q2 is as an estimate of the chance that an MM probe is cross-hybridizing to another target transcript (or that most of its signal is from non-specific hybridization). A reasonable estimate of q2 is given by subtracting this estimate of cross-hybridization potential from 100%. In the examples that follow, q2 = 90%. In practice, the value of q2 was found to have little effect on the background correction (see Figure 6). This is understandable because the probe sets have been pre-selected to avoid cross-hybridization of both the PM and MM probes.

## Conclusion

We have shown that microarray data from three different Affymetrix platforms (GoldenSpike, HG-U95Av2, and HG-U133A) do not meet the assumptions of the exponential-normal convolution model for background correction. This model is used in the Bioconductor software package in conjunction with quantile normalization and median polish summarization to comprise the RMA method. In all cases examined, estimated background noise did not follow a normal distribution, nor did the resulting estimated signal follow a simple exponential distribution. To circumvent these problems, we devised a distribution-free method to subtract background noise (DFCM). This method tended to perform better than many popular algorithms across a variety of experiments and array platforms.

This finding has four important implications. First, it is important to account for non-specific hybridization. We attempted to do so by using MM intensities to obtain an estimate of background noise. MAS 5.0 uses ideal mismatch to account for non-specific hybridization, but given the strong correlation between PM and MM values, the method is likely subtracting signal from the PM intensities, resulting in poor sensitivity and specificity. In other words, the method does not really account for non-specific hybridization, since the MM values do not perform as designed.

GCRMA uses the probe sequence information given by MM probes and it works well for the HG-U95Av2 data, but not for the HG-U133A data. The performance discrepancy may be explained in part by the improved technology and better knowledge of the human genome at the time of the creation of the HG-U133A chip. The other part of the explanation lies with deficiencies in estimating the various components of the GCRMA model. For example, parameter estimates for nonspecific hybridization are difficult to estimate reliably since the signal and noise from an observed intensity cannot be distinguished for most data. Estimating probe affinity is also quite difficult in practice. It has been reported that the top 2% probes will contain up to 50% of total signals [3]. If there are not enough arrays from enough different conditions the estimated affinity will be very biased towards probes with high intensities. In addition, the probe affinity relating to nonspecific hybridization should be investigated instead of that belonging to the whole signal since we want to know the effect of nonspecific hybridization between PM and MM within a probe pair. Therefore, it would be better to use "nonspecific hybridization" rather than the observed intensities. The difference between the observed intensity and the unknown nonspecific hybridization rate might be of practical importance.

Second, any background correction method based on assumptions that the background noise is normally distributed and that the real signal is exponentially distributed may not be valid for any given array platform. Testing the distributional assumptions for real data is impossible, since we cannot know what is background and what is signal. Third, it is clear that we need to develop an understanding of the reasons certain methods perform better on certain platforms, and the role that non-specific hybridization and cross-hybridization play in the observed intensities from microarray data. Finally, the fact that different methods perform better (or worse) on different platforms indicates that no one method may be a panacea for all preprocessing needs. However, in order to test this conjecture, more spike-in data sets on a variety of platforms are necessary, as well as performance measures for use on real data sets. Automated methods for choosing the best method to analyze a particular microarray data set would be an important contribution.

## Appendix

Please see Additional file 1

## Competing interests

The authors declare that they have no competing interests.

## Authors' contributions

ZC devised the algorithms and performed the study; MM and RHS supervised the study, obtained supports and drafted the manuscript; QL helped to design the algorithms; YD assisted in the study; MK developed and ran the code for the CLASSIFI algorithm. All authors have read and approved the final manuscript.

## Supplementary Material

Additional file 1 Click here for file
