# Supplementary material for: A distribution-free convolution model for background correction of oligonucleotide microarray data
Source: BMC Genomics. 2009 Jul 7;10(Suppl 1):S19. doi: 10.1186/1471-2164-10-S1-S19 (PMC2709262; doi:10.1186/1471-2164-10-S1-S19)
Supplement: Additional file 1 [file 1471-2164-10-S1-S19-S1.doc]

Appendix: R Codes:

## To use the following code, copy and paste it into a plain text file.

## open an R session, and source the file into R. Once the affy

## library package is loaded, type

## bgcorrect.methods=c(bgcorrect.methods, “nonpara003”)

## to add DFCM to the list of possible background correction methods.

## Then type

## expresso(data,bgcorrect.method="nonpara003",

## normalize.method="quantiles",

## pmcorrect.method="pmonly",summary.method="medianpolish")

## Here, data is an AffyBatch object.

bg.correct.nonpara003=function (object, ...)

{

## nonpara noise estmate

noise.est.nonpara=function(pm,mm,q1=0.1,q2=0.95){

max.density <- function(x, n.pts=2^12) {

aux <- density(x, kernel = "epanechnikov", n = n.pts,

na.rm = TRUE)

aux$x[order(-aux$y)[1]]

}

quan=quantile(pm,q1)

noise.data=mm[pm<quan] ### we may use some robust method to take off

the outlier

max.noise=quantile(noise.data,q2) ### use the lowest q2*100% of those

MM to estimate the noise data

noise.data=noise.data[noise.data<max.noise]

noise.mean=max.density(noise.data)

noise.se.data=noise.data[noise.data<noise.mean]-noise.mean

noise.se=sqrt(sum(noise.se.data^2)/(length(noise.se.data) - 1)) *

sqrt(2)

res=list(mean=noise.mean,se=noise.se)

return(res)

}

########

bg.adjust.nonpara003=function(pm,mm){

min=min(min(pm),min(mm)) ## minum of the intensity

para=noise.est.nonpara(pm,mm)

mean=para$mean

se=para$se

for(k in 1:length(pm))

{## (min,mean+2*se)-->(1,2*se):

if(pm[k]<mean+2*se) {pm[k]=1+(pm[k]-min)*(2*se-1)/(mean+2*se-min)}

else {pm[k]=pm[k]-mean}

}

return(pm)

}

for (i in 1:length(object))

{

pm(object)[,i]=bg.adjust.nonpara003(pm(object)[,i],mm(object)[,i])}

return(object)

}
